# Robust Localization of Low-Velocity Impacts on Honeycomb Sandwich Panels via FBG Sensor Networks

**DOI:** 10.3390/s26051715

**Published:** 2026-03-09

**Authors:** Zhengwen Zhou, Yibo Yang, Xin Xu, Kexia Peng, Yihong Han, Guangming Song, Jingtai Li, Zhe Lin, Liangjie Guo

**Affiliations:** 1 Faculty of Engineering, China University of Geosciences (Wuhan), Wuhan 430074, China; 1202110523@cug.edu.cn (Z.Z.); 1202420557@cug.edu.cn (Y.Y.); 1202420512@cug.edu.cn (Z.L.); 2 DFH Satellite Co., Ltd., Beijing 100081, China; peterxuxin@126.com (X.X.); pengkexia90@126.com (K.P.); hanyh97zju@126.com (Y.H.); 3 Beijing Institute of Spacecraft Environment Engineering, Beijing 100094, China; guangming.012@163.com (G.S.); li_jing_tai@163.com (J.L.)

**Keywords:** impact localization, template matching, fiber Bragg grating, honeycomb

## Abstract

**Highlights:**

**What are the main findings?**

- The proposed method can be applied to the localization of low-velocity impacts on honeycomb sandwich panels, and can achieve high positioning accuracy.
- The application of wavelet denoising and error outlier weighting in template matching method can improve the positioning accuracy.

**What is the implication of the main finding?**

- This work enhances the impact localization accuracy of multilayer honeycomb sandwich panels commonly used in aerospace by mitigating noise and overcoming signal attenuation, thereby enhancing flight safety and providing a structural health monitoring solution for thick multilayer structures.

**Abstract:**

Honeycomb sandwich panels are widely used in aerospace, yet they are vulnerable to low-velocity impacts. Implementing effective localization is challenging because, unlike single-layer structures, the multi-layer energy dissipation capabilities of honeycomb core induce rapid stress wave attenuation and reverberations, degrading signal quality. This paper designs a testing platform for low-velocity impact and proposes a template matching method based on wavelet denoising and error outlier weighting. This method is based on 16 FBG sensors uniformly arranged on the panel, dividing the panel into 25 × 25 grids, with five impacts in each grid forming a template library. Similarity matching is performed by calculating the Euclidean distance between the template library and test signals, combined with wavelet denoising and outlier weighting to compute the average localization accuracy. The results show that for a honeycomb panel measuring 500 mm × 500 mm × 20 mm, the basic method yields an average localization accuracy of 21.29 mm. When error outlier weighting is applied, the accuracy improves to 12.36 mm. Finally, by combining outlier weighting with Sym5 wavelet denoising, the average error is further reduced to 8.53 mm. These results demonstrate that the proposed method mitigates the effects of signal instability in honeycomb structures, providing a robust and precise solution for aerospace SHM where traditional methods fall short.

## 1. Introduction

Honeycomb sandwich panels are extensively utilized across multiple sectors [1], such as aerospace [2,3], vehicle impact resistance [4,5,6], architectural science [7,8], battery protection [9] and solar power generation [10], owing to their thermal protection properties, material efficiency, and strength advantages [11]. Due to their ability to significantly reduce weight and enhance protection, they are popular in the aerospace field, having already been used in both military and civilian aerospace vehicles, and are expected to be utilized in outer space satellites [12]. Compared to traditional aluminum composites, honeycomb sandwich structures are very sensitive to impact loads and exhibit complex damage characteristics that are difficult to detect [13]. Low-velocity impacts on aerospace composite structures, such as space debris or bird strikes, could result in barely visible impact damage (BVID) [14], potentially leading to structural failure as time progresses [15]. Therefore, structural health monitoring (SHM) is needed to locate low-velocity impacts and to assess the strength and stiffness of the impact sites through technical means, in order to minimize the risk of structural failure in spacecraft due to BVID [16].

In recent years, researchers have undertaken a variety of studies in the area of composite material impact monitoring, especially regarding low-velocity damage and impact responses of honeycomb sandwich materials, but there has been little research on the localization of low-velocity impacts in honeycomb materials. Huo et al. [17] systematically investigated the effects of various structural parameters, such as wall thickness and core height, on impact response by studying the failure and damage mechanisms of honeycomb sandwich structures. Wang et al. [18] also investigated four different damage states of honeycomb sandwich structures under various energy impacts. Ma et al. [19] investigated the damage initiation and evolution processes of honeycomb sandwich structures subjected to low-velocity impacts and compressive loads after the impact. Zhang et al. [20] examined the dynamic impact response of materials with and without honeycomb sandwich core structures subjected to lateral low-speed impacts. He et al. [21] investigated the low-speed impact response and damage characteristics of aluminum honeycomb sandwich structures featuring carbon fiber composite panels. Honeycomb structures exhibit good protective and shielding performance under ultra-high-speed impacts in aerospace applications [22].

Over the past few decades, various methods for monitoring composite material damage have been tested, including piezoelectric (PZT) sensors [23,24], ultrasonic sensors [25,26] and fiber Bragg sensors (FBG) [27,28]. FBG sensors, in particular, have gained immense traction across SHM [29,30], thermal mapping, and biomedical applications [31]. This widespread adoption is largely attributed to their compact footprint, robust multiplexing capabilities, and inherent immunity to electromagnetic noise. Furthermore, research conducted by Juwet et al. [32] verified the reliability of these optical sensors in extreme environments; their study revealed that exposing composite materials with integrated fibers to Low Earth Orbit (LEO) vacuums degrades neither the sensor functionality nor the host structure’s integrity.

For high-speed impact damage, FBG sensors can identify the impact location and damage severity by comparing with theoretical simulations [33]. Veek et al. [34] studied the real-time control of structural vibrations in scaled samples of actual launch vehicles using FBG sensors. Duan et al. [35] tested the sensitive areas of FBG and proposed three optimization schemes for FBG layouts, validating the effectiveness of collision localization through several impact tests. Yu et al. [36] established a linear acoustic emission localization method using FBG sensors, which can accurately locate actual damage sources during three-point bending tests of laminated plates. Chen et al. [37] proposed a smart sensor-enhanced method for discontinuous aerospace structures by utilizing FBG sensors to monitor the strain information of aircraft, improving the flight performance of smart aircraft. Ciminello et al. [38] validated a real-time SHM system using a modular FBG sensor network and FBG array, which can identify the “edges” of damaged areas and make predictions. Li et al. [39] proposed an innovative method based on FBG monitoring data for structural damage identification and anomaly localization, which enhances the capability of quantitative damage identification and localization. Qiu et al. [40] introduced a novel hybrid response similarity search and optimization strategy that quickly determines several potential locations and then refines the precise position of the impact load by examining the nominal residuals between the reconstructed responses at those locations and the actual responses, greatly enhancing both localization efficiency and accuracy.

Currently, there are four main methods for impact localization of composite materials based on FBG sensors. The first method is the Time Difference of Arrival (TDOA) method [41,42], which locates impacts based on the time difference of sound waves reaching the sensors combined with wave speed. Its advantages include a relatively simple algorithm and applicability to single-layer structures; however, due to the anisotropy of honeycomb sandwich panels and the complex reflection of internal stress waves, it is challenging to accurately determine the wave speed and arrival time of sound waves at the sensors, which may result in discrepancies where farther points have earlier arrival times, leading to unstable localization results. Chen et al. [43] introduced a rose connection method for FBG sensors to replace individual FBG sensors, improving the positioning accuracy of triangulation methods. The second method involves establishing a mapping relationship between the characteristic signals of FBG sensors and impact response signals, comparing reference information with test signals using various algorithms to obtain impact localization results [44,45]. However, this method imposes high requirements on experimental conditions and algorithms. Zheng et al. [46] applied a projection dictionary learning algorithm on a carbon fiber aluminum honeycomb sandwich panel of dimensions 300 mm × 300 mm × 15 mm, using structured sparse representation for collision localization, which resulted in an accuracy of 96.7% for impact localization. The third method involves modeling and locating the impact position by minimizing the differences based on the objective function of simulated signals [47]. The complexity of honeycomb sandwich structures results in non-linearity under different parameters, making accurate prediction of impact localization challenging, and random noise often leads to considerable inaccuracies during practical application of this method. The fourth method is a template matching approach based on neural networks and feature libraries, which can help mitigate the challenges posed by anisotropy and complex internal stress wave reflections in honeycomb sandwich panels. Li et al. [48] performed collision detection by embedding FBG sensors and employing an integrated learning approach. Wen et al. [49] developed a low-velocity impact monitoring and testing system for Carbon fiber reinforced plastic (CFRP) laminated plates based on FBG sensors, implementing impact localization using Fast Fourier Transform (FFT) and Principal Component Analysis (PCA) as inputs for a Back Propagation (BP) neural network model, resulting in an average localization error of 2.1 cm. Shrestha et al. [50] conducted low-velocity impact monitoring using an error outlier localization method, achieving a localization error of 10.7 mm on composite plates. Huan et al. [51] utilized FBG sensors affixed to the surface of carbon fiber composite tubes to record collision signals from various positions, while also applying a BP neural network and an outlier-based error method for localization. Their research concluded that the outlier-based error method offers greater computational accuracy but necessitates a substantial dataset.

However, despite the progress in FBG-based low-velocity impact localization, several challenges remain, particularly regarding honeycomb sandwich structures:

(1) Lack of Full-Field Experimental Validation: Most existing studies rely on sparse sampling or limited impact points. Research that conducts comprehensive localization testing covering the entire surface of honeycomb panels to systematically evaluate algorithm robustness across different regions is still scarce.

(2) Signal Complexity and Noise: Impact signals in honeycomb structures exhibit high self-similarity and are susceptible to significant noise interference due to complex internal reflections. This necessitates robust signal processing techniques to more reliably extract valid features from the noisy background.

To mitigate these challenges, this paper proposes a robust impact localization framework for honeycomb sandwich panels using a distributed FBG sensor network. This method integrates Sym5 wavelet denoising to reduce structural noise and an error outlier-weighted template matching algorithm to limit the influence of anomalous data points, thereby improving overall localization accuracy. The remainder of this paper is organized as follows: Section 2 introduces the fundamental principles of FBG sensors and validates the effectiveness of the wavelet denoising method. Section 3 details the experimental setup, sensor layout, and impact testing procedure. Section 4 analyzes the characteristics of impact signals and elaborates on the implementation of the outlier-weighted template matching algorithm. Section 5 presents the localization results, discusses the error distribution, and benchmarks the performance against the existing literature. Finally, Section 6 concludes the study.

## 2. Theoretical Principles

### 2.1. Operating Mechanism of FBG Sensors

**Figure 1:**
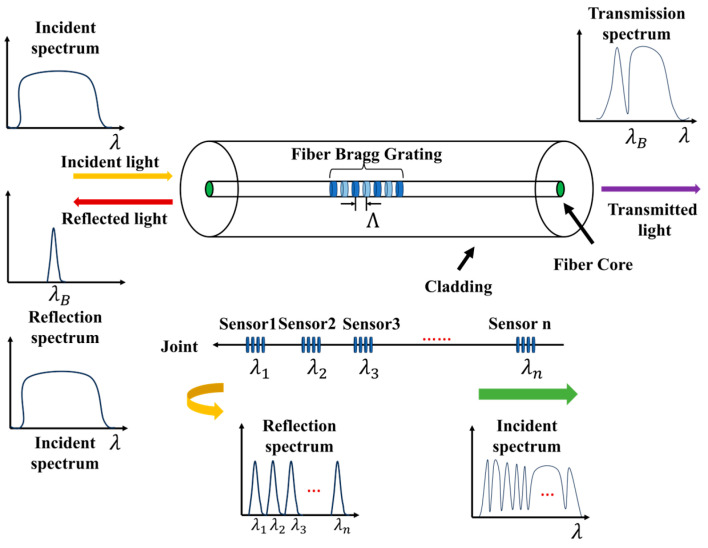
The schematic diagram of FBG illustrates the input broadband optical spectrum signal, the Bragg grating reflection signal, and the optical fiber core transmission signal.

Functioning as wavelength-modulated devices, FBG sensors capture environmental variations by tracking shifts in the Bragg wavelength. As broadband incident light travels through the optical core, the grating specifically reflects a narrow band of wavelengths that matches the Bragg condition. Meanwhile, the remaining unmatched light spectrum propagates through the fiber without disruption [52]. This reflected optical signal determines the center wavelength, a process summarized in Figure 1.

According to the theory of fiber coupling modes, when an incident light wave propagates in the FBG, mode coupling occurs, and its center wavelength can be expressed as:

(1) $$\lambda_{B}=2n_{eff}\Lambda$$

where $\lambda_{B}$ represents the center wavelength of the FBG sensor, $n_{eff}$ denotes the effective refractive index of the fiber core, and Λ denotes the period length of the grating.

Taking the total differential of Equation (1) yields:

(2) $$\Delta \lambda_{B}=2\Delta n_{eff}\Lambda +2n_{eff}\Delta \Lambda$$

Equation (2) indicates that the period of the fiber grating or the effective refractive index will cause a shift in the center wavelength of the FBG. Considering only the axial stress on the fiber grating while keeping the temperature field constant, based on the following assumptions:

(1) The structure is simplified to two layers: cladding and core;
(2) The optical fiber follows Hooke’s law and does not experience shear stress;
(3) The variation in the photosensitive refractive index of the core is uniformly distributed across the cross-section, which does not affect the isotropic properties of the fiber;
(4) All stresses are static stresses.

From Equations (1) and (2), it can be concluded that:

(3) $$\Delta \lambda_{B}=2\left[\frac{{\partial n}_{eff}}{\partial L}\Delta L+\frac{{\partial n}_{eff}}{\partial a}\Delta a\right]\Lambda +2n_{eff}\frac{\partial \Lambda }{\partial L}\Delta L$$

where ΔL represents the axial elongation of the fiber grating, Δa indicates the change in the fiber diameter caused by axial tension, $\frac{{\partial n}_{eff}}{\partial L}$ refers to the elasto-optic effect, and $\frac{{\partial n}_{eff}}{\partial a}$ pertains to the waveguide effect.

The relative dielectric permittivity tensor ($\beta_{ij}$) is related to the refractive index ($n_{ij}$) in a certain direction as follows:

(4) $$\beta_{ij}=\frac{1}{{n_{ij}}^{2}}$$

Based on the isotropy of the fiber grating, the $n_{ij}$ in all directions is the same. Therefore, $n_{eff}$ can be used to replace $n_{ij}$ for differentiation. When external stress is present, $\beta_{ij}$ can be expressed as a stress function. Introducing the elasto-optic coefficient ($p_{ij}$) and performing a Taylor series expansion while neglecting higher-order terms, we obtain:

(5) $$∆\beta_{ij}=∆\left(\frac{1}{{n_{ij}}^{2}}\right)=\left(p_{11}+p_{12}\right)\varepsilon_{r}+p_{12}\varepsilon_{z}$$

where $\varepsilon_{z}=\frac{∆L}{L}$ is the axial elongation strain, $\varepsilon_{r}=-v\varepsilon_{z}$, and v denotes the Poisson’s ratio of the fiber.

When subjected to homogeneous tensile deformation, the standard optical fiber conforms to the relationship given in Equation (6).

(6) $$\frac{\partial \Lambda }{\Lambda }\times \frac{L}{\partial L}=1$$

Consequently, the shift in the grating’s Bragg wavelength driven purely by axial strain can be quantified as follows [53]:

(7) $$\frac{\Delta \lambda_{B}}{\lambda_{B}}=\left\{1-\frac{{n_{eff}}^{2}}{2}\left[p_{12}-(p_{11}+p_{12})v\right]\right\}\varepsilon_{z}=\left(1-p_{e}\right)\varepsilon_{z}={S_{e}\varepsilon }_{z}$$

In this context, $p_{e}=\frac{{n_{eff}}^{2}}{2}\left[p_{12}-(p_{11}+p_{12})v\right]$ denotes the effective photoelastic constant, while $S_{e}$ acts as the strain sensitivity factor of the fiber grating, $S_{e}=1-P_{e}$.

The equation indicates that there is a certain linear relationship between the central wavelength shift of the FBG sensor and strain. When the honeycomb structure undergoes micro-deformation due to impact, it causes a shift in the FBG wavelength bonded to the structural surface [54]. Therefore, FBG sensors can be used in the research of monitoring low-velocity impact locations.

### 2.2. Denoising Principles of Sym5 Wavelet Transform

In low velocity impact positioning experiments, the signals collected by the FBG sensor are often affected by a large amount of environmental noise due to the difficulty in isolating external interference, which reduces the signal-to-noise ratio. These noise signals can have a significant impact on the accuracy of the impact positioning, potentially leading to inaccurate positioning results. Wavelet transform can decompose high-frequency signals downward, ultimately obtaining the components of each frequency domain [55].

Wavelet threshold denoising is a noise reduction technique based on wavelet orthogonal decomposition, which involves decomposing the original signal using wavelet transformation to obtain wavelet coefficients, followed by threshold processing to retain coefficients, and finally performing wavelet reconstruction [56]. When selecting a wavelet basis function, Symlets (Sym) wavelets are chosen due to their orthogonality, compact support, and approximate symmetry, making them nearly symmetric compactly supported orthogonal wavelets. Compared to Daubechies (db) wavelet functions, it has been improved with a support range of 2N−1 and a vanishing moment of N, demonstrating better regularity and superior symmetry compared to the db function. This helps to reduce phase distortion during signal analysis and reconstruction to some extent, leading to the selection of the Sym5 wavelet basis function in this paper [57].

The core of wavelet transform lie in decomposing the signal f(t) into components of different frequencies, thereby enabling multi-scale analysis of the signal [58]. The wavelet transform can be expressed as:

(8) $$W_{f}\left(a,b\right)=\int_{-\infty }^{\infty }f(t)\psi_{a,b}(t)dt$$

where $\psi_{a,b}(t)$ refers to the wavelet function, a is the scale factor, and b is the translation factor. The procedure for wavelet denoising is as follows:

First, perform wavelet transform on the noisy signal f(t) to obtain a set of wavelet coefficients, $W_{f}\left(a,b\right)$;

Secondly, by threshold processing $W_{f}\left(a,b\right)$, the estimated wavelet coefficient ${\hat{W}}_{f}\left(a,b\right)$ is obtained, so that the $\left\|{\hat{W}}_{f}\left(a,b\right)-{\hat{\mu }}_{f}\left(a,b\right)\right\|$ is as small as possible;

Lastly, wavelet reconstruction is carried out by using ${\hat{W}}_{f}\left(a,b\right)$ to obtain the estimated signal ($\hat{f}(t)$), which is the signal after denoising.

The hard thresholding method sets a threshold ($\lambda =\sigma \sqrt{2lg(N)}$), retaining the coefficients whose absolute values are greater than the threshold. The formula is as follows:

(9) $${\hat{W}}_{f}\left(a,b\right)=\{\begin{array}{c} W_{f}\left(a,b\right),\;\;if\;\left|W_{f}\left(a,b\right)\right|>\;\lambda \;\\ 0,\;\;\;\;\;\;\;\;\;\;\;\;\;\;\;\;\;\;\;\;\;\;\;otherwise \end{array}$$

The advantage of the hard threshold method lies in its ability to effectively preserve the high-frequency components of the signal. However, its disadvantage may lead to spikes in the signal, resulting in noticeable distortion during signal reconstruction.

The soft threshold method [59] not only preserves the coefficients that exceed the threshold, but also reduces these coefficients, thereby generating a smoother signal. The formula can be expressed as:

(10) $${\hat{W}}_{f}\left(a,b\right)=\{\begin{array}{c} W_{f}\left(a,b\right)-\lambda sign(W_{f}\left(a,b\right)),\;\;if\;\left|W_{f}\left(a,b\right)\right|>\;\lambda \;\\ \;\;\;\;\;\;\;\;\;\;\;\;\;\;\;0,\;\;\;\;\;\;\;\;\;\;\;\;\;\;\;\;\;\;\;\;\;\;\;\;\;\;\;\;\;\;\;\;\;\;otherwise \end{array}$$

The benefit of the soft threshold method is that it diminishes the effect of noise on the signal, while also somewhat decreasing phase distortion. However, this can lead to a decline in the overall amplitude of the signal. The existing literature compares the differences between the two by simulating white noise using computer models [60].

To validate the feasibility of the thresholding methods under realistic engineering conditions, an actual impact response signal collected by the FBG sensors on the 20 mm honeycomb sandwich panel was utilized, replacing the conventional simulated white noise approach. This is crucial because cellular structures generate highly specific noise distributions due to their complex multipath reverberation, mode conversion, and high-frequency acoustic scattering from internal cell walls, which cannot be replicated by standard Gaussian white noise models.

**Figure 2:**
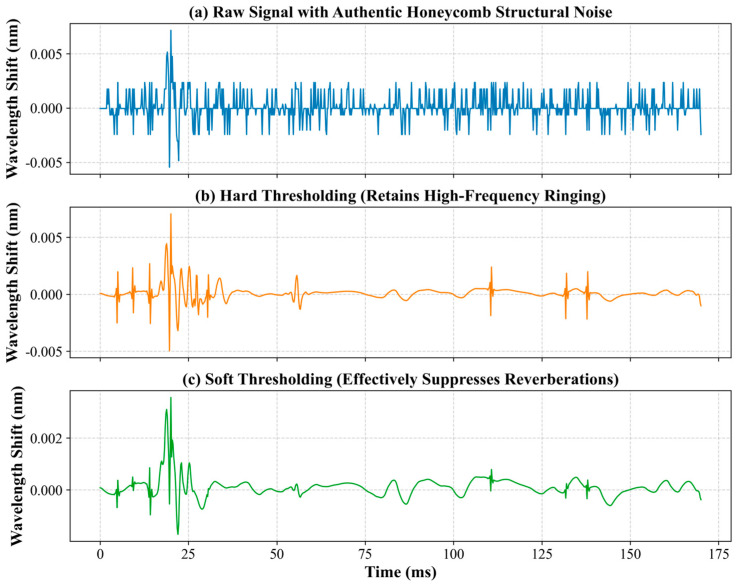
Comparison of Sym5 wavelet denoising techniques applied to an authentic honeycomb structural impact signal: (**a**) the raw FBG sensor signal contaminated with multi-path structural reverberations; (**b**) the signal reconstructed using hard thresholding, which preserves the main peak but retains sharp high-frequency residual spikes (ringing); and (**c**) the signal reconstructed using soft thresholding, demonstrating effective suppression of complex reverberations while maintaining a highly smooth and stable baseline.

Figure 2 illustrates the raw FBG signal contaminated by this authentic structural noise, alongside the reconstructed signals processed via both soft and hard thresholding methods using the Sym5 wavelet. As shown in the figure, while hard thresholding retains some high-frequency structural ringing, the soft thresholding method suppresses the complex honeycomb reverberations. Furthermore, the signal reconstructed by the soft thresholding method maintains a smooth baseline and defines the primary impact peaks without inducing significant phase distortion. Consequently, the Sym5 wavelet combined with soft thresholding was selected as the optimal denoising strategy for this study.

**Figure 3:**
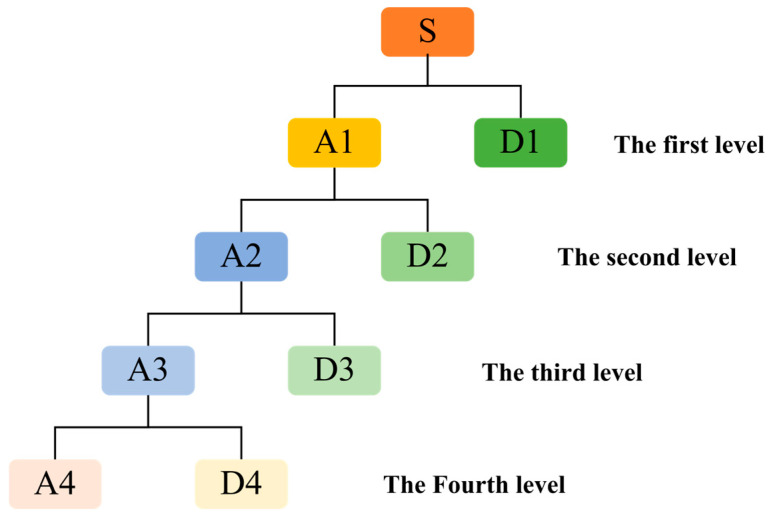
Diagram of the 4−level Sym5 wavelet decomposition process used for signal denoising. In this hierarchical structure, ‘S’ represents the original raw impact signal. ‘A1’ through ‘A4’ denote the Approximation coefficients, which capture the low−frequency primary components of the stress wave at each consecutive level. Conversely, ‘D1’ through ‘D4’ represent the Detail coefficients, which isolate the high-frequency structural noise and interference targeted for thresholding.

Furthermore, the number of decomposition levels is important for wavelet transformation. This paper processes the original impact signals from the FBG sensor using Sym5 wavelet basis functions at various levels, using the mean squared error and signal-to-noise ratio after denoising as metrics for determining the level, ultimately selecting a decomposition level of 4 [51]. Figure 3 illustrates the process of Sym5 wavelet decomposition at a decomposition level of 4.

**Figure 4:**
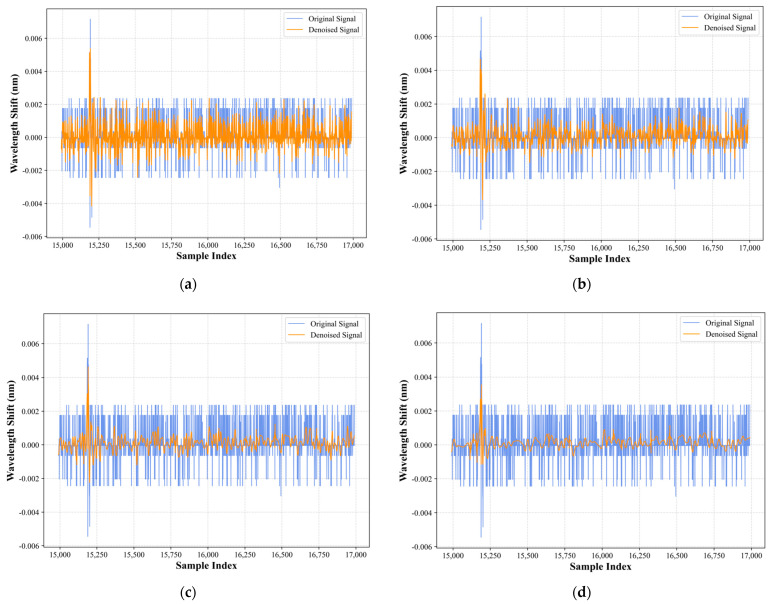
Comparison between the collision signals received by the FBG sensor at different decomposition levels using the Sym5 wavelet soft threshold denoising method: (**a**) 1-level decomposition; (**b**) 2-level decomposition; (**c**) 3-level decomposition; and (**d**) 4-level decomposition.

**Figure 5:**
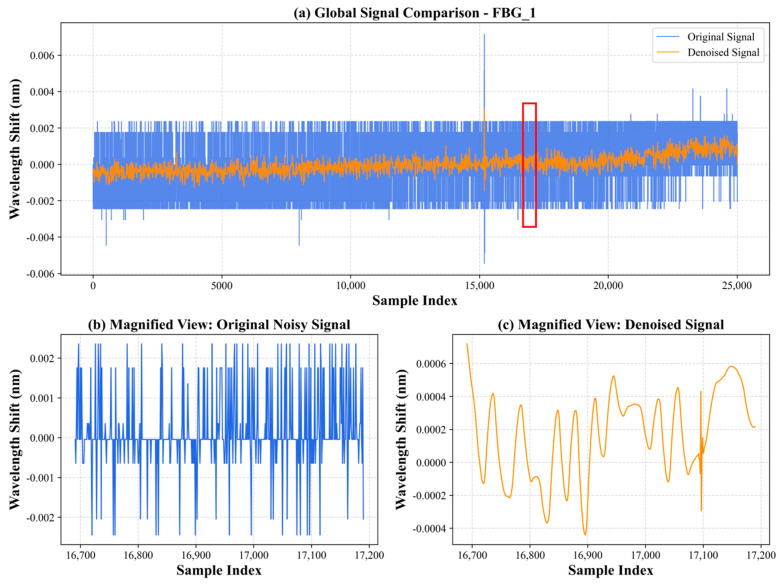
Comparison of the FBG sensor response signal before and after Sym5 wavelet soft-threshold denoising. (**a**) The global signal overlay, where the blue line indicates the raw noisy signal and the orange line indicates the denoised signal; the red box highlights the 500-sample window extracted for magnification. (**b**) The magnified view of the original signal, displaying high-frequency fluctuations. (**c**) The magnified view of the smoothed denoised signal. Specific minimum and maximum wavelength shift levels have been added to the y-axes of both insets to quantitatively illustrate the reduction in noise. The red box in the image represents the sampling location.

Figure 4 illustrates the variation in the signal when applying the Sym5 wavelet basis for soft threshold denoising with different decomposition levels (1 to 4 levels). Comparative analysis shows that as the number of wavelet decomposition levels increases, the denoising effect is gradually enhanced. From (a) to (d), the difference between the denoised signal (orange line) and the original signal (blue line) in the high-frequency fluctuations gradually widens, indicating more effective suppression of noise components. Especially in the four-level decomposition shown in (d), the noise is almost completely filtered out, the signal becomes smoother, the stability of the denoised signal is further improved, and random fluctuations are reduced. Furthermore, despite the increase in decomposition levels, the main peak of the impact signal is still well retained without serious distortion of the main signal. This indicates that Sym5 four-level soft threshold denoising maintains good signal fidelity, improves the signal-to-noise ratio, and provides a solid foundation for subsequent impact localization. Figure 5 shows the signals processed with the Sym5 wavelet soft threshold denoising method at 4 decomposition levels, along with the original signal and a magnified view of 500 sampling units of the denoised signal. The blue signal represents the original signal, while the orange signal represents the denoised signal. From the comparison analysis, it can be observed that the wavelet-transformed signal is smoother and retains the complete main peak of the signal.

## 3. Experimental Setup and Process

### 3.1. Low Velocity Impact Monitoring System

**Figure 6:**
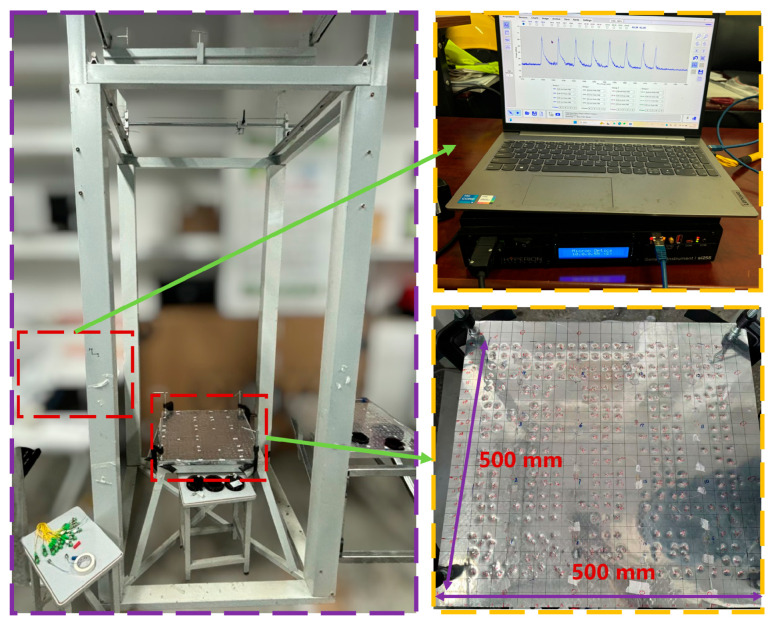
The low-velocity drop ball impact test platform, the Fiber Bragg Grating demodulator, and the honeycomb structure panel with FBG sensors arranged (500 mm × 500 mm × 20 mm).

To conduct low-speed impact tests on honeycomb structure panels, a low-velocity drop ball impact test platform was designed at China University of Geosciences (Wuhan), as shown in Figure 6. The platform includes a drop ball device and a fastening device, utilizing an electromagnetic mechanism to adjust the initial drop velocity. Additionally, the stability of the honeycomb structure panel is ensured by a rubber seal on the clamping device, while the position of the falling ball is controlled by a sliding block above. The signals during the impact process are collected using FBG sensors, and the signals are demodulated using a Fiber Bragg Grating demodulator (model: si 255), which has 16 channels and a sampling frequency of 5000 Hz. The impacted honeycomb structure panel measures 500 mm × 500 mm × 20 mm, with 16 FBG sensors evenly distributed across its structure, as shown in Figure 6.

### 3.2. Procedure for Low Velocity Impact Tests

Firstly, the honeycomb structure panel measuring 500 mm × 500 mm × 20 mm is clamped onto the low-velocity drop ball impact test platform, and the panel is divided into a grid area of 25 × 25 grids, with each grid area measuring 20 mm × 20 mm, while the origin of the coordinate axes is marked in the lower left corner, allowing any impact point to be represented by coordinate values and grid indices. To prevent effects of the tested structure from causing scattering of the shock wave and energy absorption that could interfere with the test system, the 40 mm edge area is supported and secured with steel bolts. The central area of 420 mm × 420 mm is selected as the impact zone.

Secondly, a 4 × 4 networks of FBG sensors are deployed on the honeycomb structure panel, totaling 16 FBG sensors, all arranged vertically and adhered to the surface of the panel using resin glue. Additionally, to prevent damage to the optical fibers upon impact, the fibers are organized through a bundling system and connected to the FBG demodulator. The demodulator is configured with a sampling frequency of 5000 Hz, and the wavelength distribution and calibration of the optical fibers have been completed. The demodulator demodulates the wavelength signals, then captures and stores the wavelength time-domain variation curves of the entire impact process, and further analysis and processing are conducted using the demodulator program on the computer.

**Figure 7:**
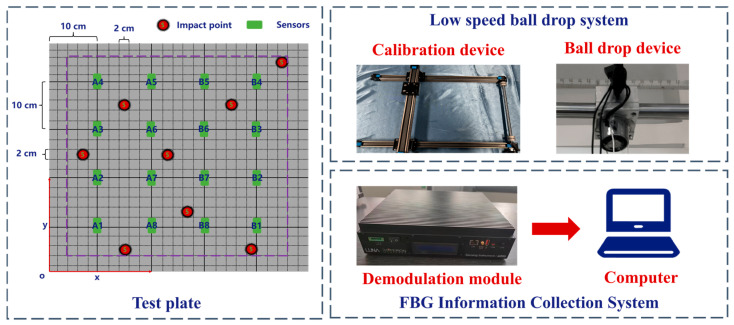
Schematic diagram of impact localization test experiment. Among them, A1–A8 and B1–B8 are all FBG sensors, set in groups of 8.

Finally, a steel ball with a diameter of 10 mm is electromagnetically adsorbed on the connection seat and is released freely from a height of 2 m by cutting off the power. The calibration device can be adjusted to move left and right, allowing collisions to occur at any position within the central area of 420 mm × 420 mm. Each grid is subjected to five impacts at the same energy level, and impact signals are recorded using FBG sensors, with the coordinates of the impact points manually documented as a template matching library. Moreover, an extra shock was applied to each grid within the area, with the shock coordinates and shock signals recorded as test signals, totaling 317 test signals collected. Figure 7 depicts the procedure, principles, and equipment utilized in the impact positioning test.

## 4. Template Matching Method Based on Error Outliers

### 4.1. Comparison of Impact Signals

**Figure 8:**
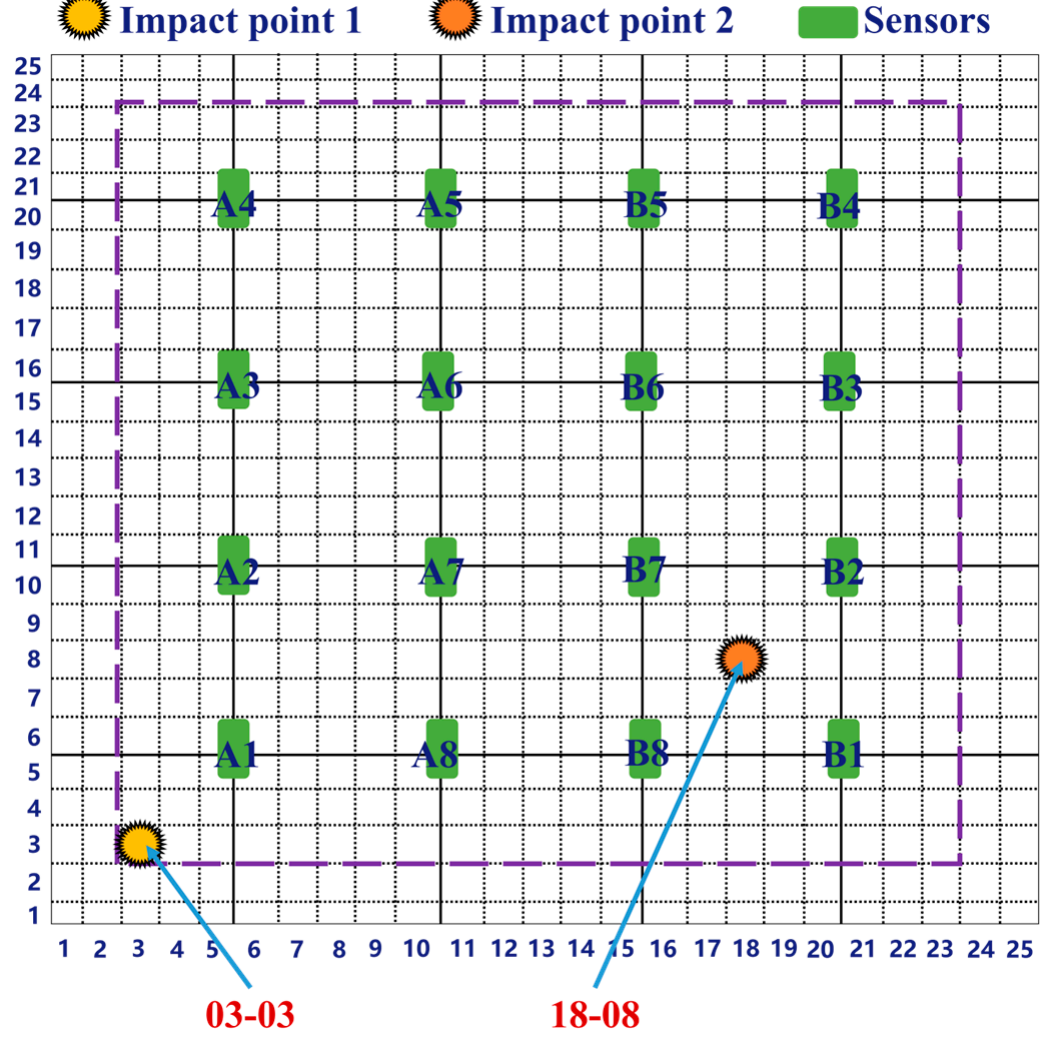
Schematic diagram of impact points and sensor layout. Among them, A1–A8 and B1–B8 are all FBG sensors, set in groups of 8.

By comparing impact signals, the differences between similar and dissimilar signals can be better understood, which helps clarify the principles of the template matching method used. As shown in Figure 8, the 500 mm × 500 mm area is divided into 25 × 25 grids, each measuring 20 mm × 20 mm. Each grid is assigned a number: the grid in the first row and first column is labeled 01-01, the grid in the second row and first column is labeled 02-01, and so on. Additionally, a central area of 420 mm × 420 mm is defined as the impact zone (indicated by the purple dashed area in the figure), where five impacts will be performed. The green squares represent the FBG sensors and their corresponding numbers. Impact point 1 is 03-03, impact point 2 is 18-08.

**Figure 9:**
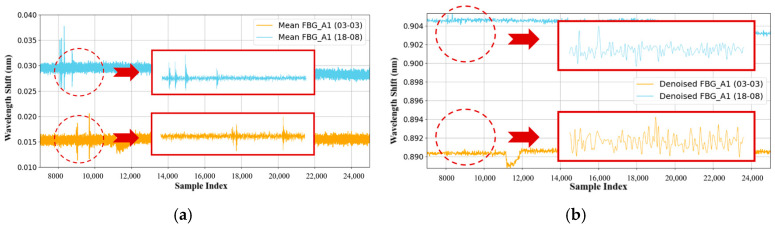
FBG_A1 received the comparison between the signals from impact point 1 and impact point 2. (**a**) Comparison of the impact signals before denoising and the enlarged comparison of the impact signals from 7000 to 10,000 ms before denoising; (**b**) Comparison of impact signals following denoising and the amplified comparison of the signals from 7000 to 10,000 ms after denoising. In both panels, the red dashed circles indicate the specific local signal range (from 7000 to 10,000), and the red arrows point to the solid red boxes, which display the magnified views of these corresponding regions to clearly illustrate the signal details.

To compare the differences between the signals of impact point 1 (03-03) and impact point 2 (18-08), FBG_A1 was chosen as the signal acquisition point to observe the differences between the two sets of signals before and after denoising. As seen in Figure 9a, the signals before and after denoising resulting from different impact locations are distinct, with considerable differences between them. Although impact point 2 is farther from the FBG_A1 sensor, its propagation speed is faster than that of the closer signal from impact point 1, and the complexity of the signal from impact point 2, along with its shock wave peaks, is much greater than that of impact point 1. This difference is attributed to the heterogeneous characteristics of the honeycomb sandwich structure plate, which lead to variations in signal echo speed and waveform count. Figure 9b shows that both methods effectively ensure the continuity of the signal, and the denoised signal exhibits distinct peaks that align with the original signal before denoising.

### 4.2. Calculation of Normalized Error Outliers

By subtracting the reference signal from the two signals mentioned above, the error value can be obtained. If the signal and the reference signal are similar, the error value will be small. By setting an appropriate dynamic threshold and comparing it with the error value, the similarity between two groups of signals can be determined. The error calculation formula is as follows:

(11) $$error\left(t_{n,i}\right)=\left|S_{ref}\left(t_{n,i}\right)-S_{test}\left(t_{n,i}\right)\right|$$

(12) $${error}_{mean}=\frac{1}{N}\sum_{i=1}^{N}error\left(t_{n,i}\right)$$

where $error\left(t_{n,i}\right)$ represents the error of the i-th signal, $S_{test}\left(t_{n,i}\right)$ denotes the denoised signal, $S_{ref}\left(t_{n,i}\right)$ refers to the reference signal, N indicates the number of signals, and ${error}_{mean}$ is the average value of the signals. After computing the average error, the Min-max normalization method scales the error to the range of [0, 1] for normalization:

(13) $${error}^{\prime }=\frac{{error}_{mean}-{error}_{min}}{{error}_{max}-{error}_{min}}$$

where ${error}_{mean}$ is the average value of the signals, ${error}^{\prime }$ is the normalized error, while ${error}_{max}$ and ${error}_{min}$ are the minimum and maximum values of the error.

Z-score normalization is used to identify outliers in the errors, which is achieved by measuring the deviation of each error data point from the overall mean of the errors, with an absolute Z-score greater than 3 defined as the criterion for identifying outliers [61].

(14) $$Z_{i}=\frac{{error}^{\prime }-\mu }{\sigma }$$

where $Z_{i}$ represents the Z-score of the error value, indicating the number of standard deviations by which the error value differs from the mean; σ denotes the standard deviation of the sample data, and μ indicates the mean of the sample data.

**Figure 10:**
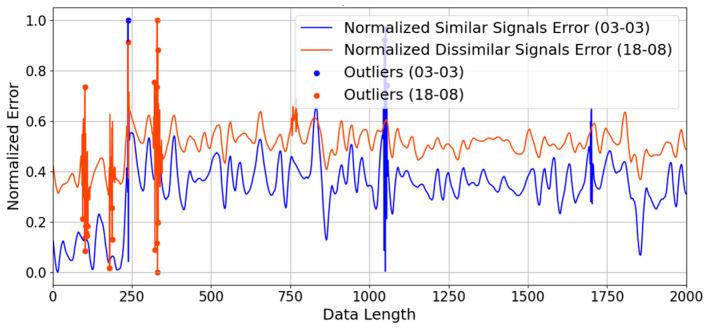
Error signal comparison between similar and dissimilar signals.

Figure 10 illustrates the point-by-point normalized error between a test signal and templates of similar (03-03, blue line) and dissimilar (18-08, orange line) impact locations across a data sequence of 2000 points. The *y*-axis represents the dimensionless Normalized Error, bounded between 0 and 1, rather than physical localization error. As observed, the error level for the dissimilar signals fluctuates around a baseline of 0.5. In this normalized context, 0.5 represents a significantly large mismatch between unrelated structural responses. In contrast, the similar signals maintain a visibly lower baseline error (approximately 0.2 to 0.4).

Two conclusions can be drawn from Figure 10. Firstly, it demonstrates the high discriminative power of the extracted features, as the correct spatial template consistently yields a lower baseline error. Secondly, it justifies the necessity of the proposed outlier weighting strategy. The plot reveals that even closely matching signals exhibit localized, extreme error spikes (marked as “Outliers” approaching 1.0). These spikes are induced by complex high-frequency ringing or slight temporal misalignments inherent to honeycomb structures. Without applying an outlier weighting penalty, these sudden, massive spikes would disproportionately dominate a standard global Euclidean distance calculation, leading to localization failures. Therefore, Figure 10 validates the algorithmic requirement to mathematically identify and suppress these outliers, ensuring that the final similarity score reflects the true baseline matching quality.

### 4.3. Impact Localization Principle Based on Template Matching

Initially, a low-velocity drop ball impact test rig is used to traverse and impact each grid in the 420 mm × 420 mm area of the honeycomb sandwich panel, with each grid being impacted 5 times to acquire reference signals. The time-domain signals of the impact collected by the FBG sensors are extracted for the first 10 ms and the following 100 ms. To eliminate matching errors caused by trigger delays and phase mismatches, a dynamic peak anchoring strategy is implemented in this study. Rather than using a fixed global trigger, the algorithm dynamically detects the absolute maximum peak ($t_{peak}$) of the denoised stress wave for each individual sensor. The 110 ms signal segment (10 ms pre-peak and 100 ms post-peak) is extracted using $t_{peak}$ as the alignment anchor, ensuring that the primary wave features of both the templates and test signals are phase-aligned. Furthermore, a temporal consistency check is applied. The peak time of each sensor is compared against the median peak time of the network. Sensors with a temporal deviation exceeding 0.2 s are contaminated by delayed reverberations and are excluded prior to the similarity matching. Environmental noise is filtered using a sym5 wavelet denoising method with 4 levels, while all signals are normalized using Min-max to obtain effective feature signals. Outliers in the impact signals are calculated using Z-score, and a database of impact locations and wavelength time-domain feature signals is established. The test signals are extracted and processed using the same method.

Then, the similarity of the signals is determined by calculating the Euclidean distance between the signals in the feature library and the unknown impact signals; the more similar the signals, the closer the impact location [62]. The calculation of Euclidean distance can be expressed as follows:

(15) $$d\left(A,\;B\right)=\sqrt{\sum_{i=1}^{n}{\left(b_{i}-a_{i}\right)}^{2}}$$

The closer the distance, the smaller d(A, B) indicates that the two signals are more similar. It is essential to highlight that the proposed matching algorithm is designed to be robust against both signal amplitude scaling and temporal shifts. Variations in impact energy inherently scale the raw signal amplitude, which could skew unnormalized Euclidean distance calculations. To ensure robustness to amplitude scaling, a Z-score normalization is applied to each extracted signal segment prior to template matching. This normalization transforms the signal to a zero mean and unit variance, eliminating the influence of absolute impact energy and preserving the unique morphological characteristics of the stress wave. Furthermore, the system is robust to temporal shifts and trigger delays due to the aforementioned dynamic peak anchoring strategy. By auto-aligning the extraction window (10 ms pre-peak to 100 ms post-peak) to the localized maximum peak of each sensor, the algorithm neutralizes uniform time-domain translations. These two pre-processing steps collectively ensure that the template matching remains stable regardless of fluctuations in impact intensity or phase timing.

The initial position $P_{i}(x_{i},\;y_{i})$ is generated based on the similarity matching results, and the inverse of the outlier values is used as weights ($w_{i}$) to perform a weighted average of the similarity matching results.

(16) $$P_{global}=(\frac{\sum_{i=1}^{N}w_{i}x_{i}}{\sum_{i=1}^{N}w_{i}},\frac{\sum_{i=1}^{N}w_{i}y_{i}}{\sum_{i=1}^{N}w_{i}})$$

(17) $$w_{i}=\frac{1}{Z_{i}+\epsilon }$$

where, $P_{global}$ is the weighted average global impact position obtained after weighting, $w_{i}$ is the reciprocal of the outlier value, and ϵ > 0 is an infinitesimally small positive number used to avoid division by zero.

Finally, based on the principle of spatial consistency, calculate the distance between the impact position corresponding to the template matching result of each sensor signal and the computed global impact position.

(18) $$d_{i}=\sqrt{{\left(x_{i}-x_{global}\right)}^{2}+{\left(y_{i}-y_{global}\right)}^{2}}$$

where $d_{i}$ is the distance between each predicted result point from the template matching and the global impact position, $x_{i}$ is the predicted x-coordinate of each template matching result, $x_{global}$ is the x-coordinate of the global position, $y_{i}$ is the predicted y-coordinate of each template matching result, and $y_{global}$ is the y-coordinate of the global position. Calculate the dynamic spatial threshold based on the median distance.

(19) $$T_{spatial}=median\left(\left\{d_{i}\right\}\right)\cdot \alpha ,\;\;\alpha >1$$

where $T_{spatial}$ is the dynamic spatial threshold, and α is the adjustment coefficient.

**Figure 11:**
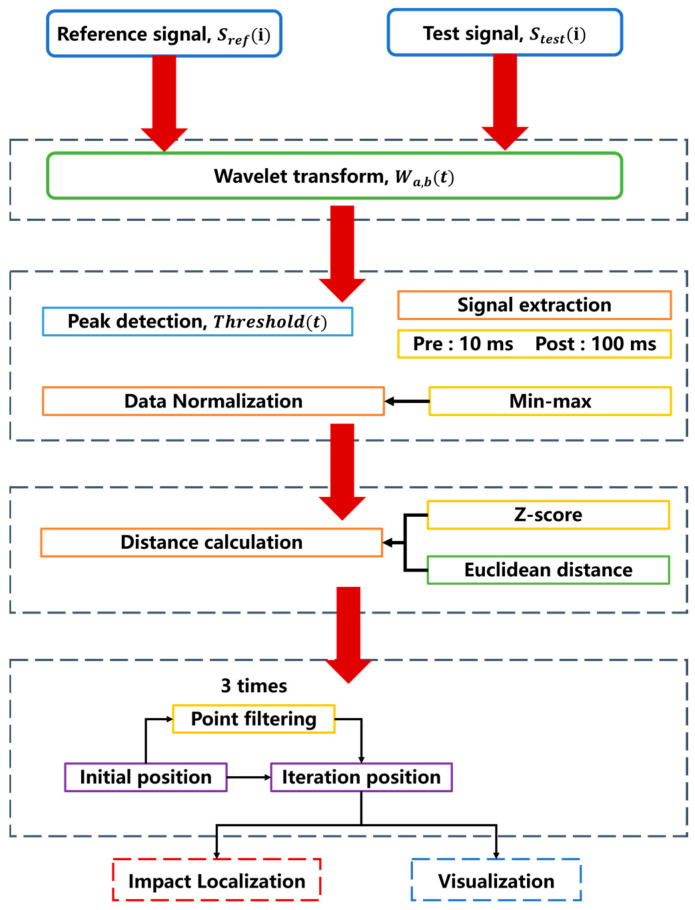
Flowchart of the template matching method based on the combination of error outliers and denoising algorithms.

Recalculate the global impact position by performing a weighted average based on the impact point positions corresponding to the template matching results of the retained sensor signals. Repeat the above steps three times until all sensors meet the consistency requirement, and determine the final computed global impact position as the predicted impact position from the template matching. To clearly summarize the entire proposed impact localization framework, Figure 11 presents a comprehensive flowchart of the methodology. It illustrates the sequential integration of the data preprocessing, Sym5 wavelet denoising, dynamic peak anchoring, distance calculation, and the final outlier-weighted spatial iteration process.

## 5. Results and Discussion

### 5.1. Results of Impact Localization

To demonstrate the spatial distribution of localization errors and the step-by-step effectiveness of the proposed framework, a representative spatial subset of 30 impact test points is presented as a visual case study.

**Figure 12:**
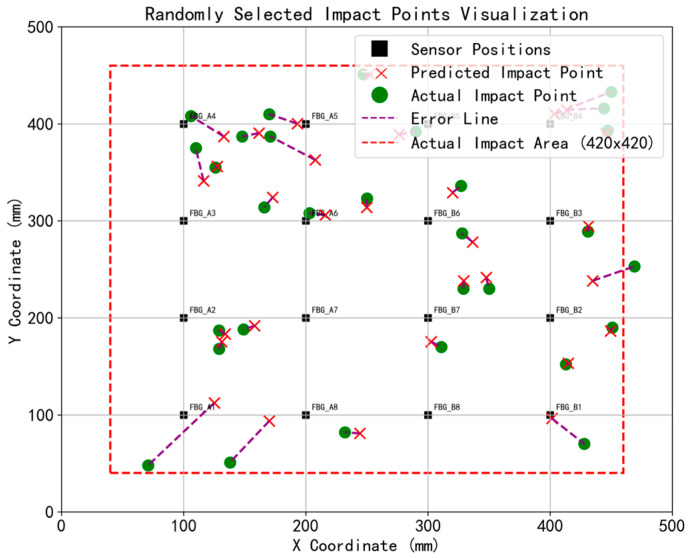
Results of impact localization without outlier weighting (30 impact cases).

Figure 12 shows the localization results calculated using only the Euclidean distance method, without the application of error outlier and denoising methods. By comparing the predicted positions and actual positions of the 30 tested points in Figure 12, it can be observed that without the error outlier method, the predicted points are biased toward the inner side of the sensor arrangement range. This inward tendency is driven by a combination of physical structural effects and the mechanics of the unweighted matching process. In terms of sensors, impacts near the boundaries suffer from complex edge reflections and signal attenuation, whereas FBG sensors capture clearer, higher-fidelity responses within their elliptical sensitive areas in the central region [35]. In terms of algorithms, when using basic unweighted Euclidean distance for template matching, the algorithm suffers from the classic boundary effect widely documented in sensor network localization [63,64]. Because edge impacts lack surrounding reference templates on their outer perimeter, the unweighted spatial averaging mathematically drags the predicted coordinates inward. This inherent bias explicitly highlights the critical necessity of introducing the error outlier weighting method.

**Figure 13:**
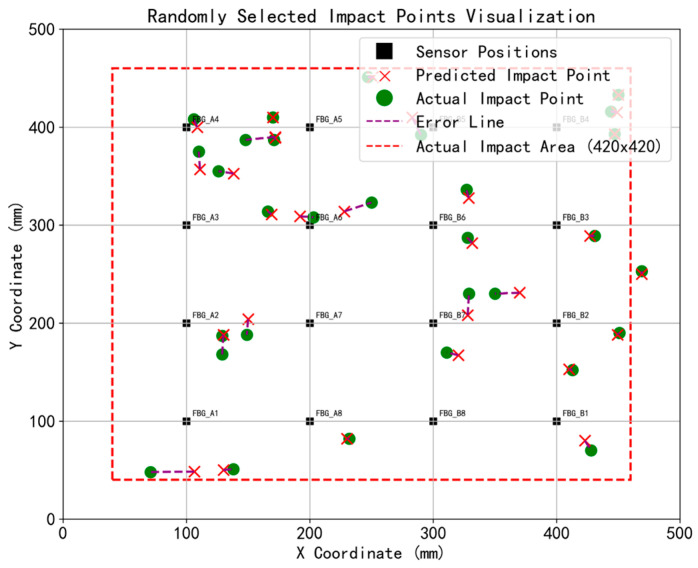
Results of impact localization without wavelet denoising (30 impact cases).

**Figure 14:**
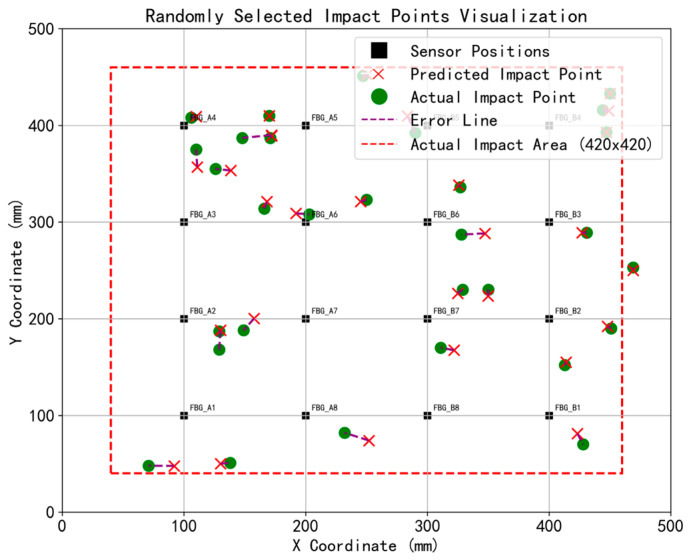
Results of impact localization with denoising and outlier weighting (30 impact cases).

Figure 13 and Figure 14 illustrate the spatial localization results achieved with the outlier-weighted method, both prior to and following Sym5 wavelet denoising, respectively. As shown in Figure 13, by heavily penalizing the anomalous edge signals, the proposed weighting strategy effectively neutralizes the inward drag, allowing the predicted points to correctly distribute towards the boundary edges. By comparing the 30 impact test points before and after denoising, it was found that the localization results before and after wavelet denoising were quite similar, with no significant change in the predicted point positions. However, wavelet denoising effectively reduced the error distance between the predicted points and the actual points, demonstrating that wavelet denoising can clearly preserve the effective information of the impact signals.

### 5.2. Analysis of Impact Positioning Results

To evaluate the statistical robustness of the proposed framework and justify the selection of the similarity metric, an expanded datasets of 317 impact points was analyzed. Table 1 presents a statistical comparison—including standard deviation, variance, and 95% Confidence Intervals (CI)—across five different distance and similarity algorithms.

To isolate the effects of the proposed algorithms, an ablation analysis was conducted. Before applying outlier weighting, the impact template matching using basic Euclidean distance yielded an average error of 21.29 mm, displaying significant localization variance (293.32 mm^2^). When outlier weighting is implemented without noise reduction, the average error substantially reduces to 12.36 mm. Ultimately, by combining outlier weighting with Sym5 wavelet denoising, the proposed Euclidean distance metric achieves an average error of 8.53 mm, with a standard deviation of 8.25 mm and a 95% confidence interval of [7.62, 9.44] mm. To validate the statistical significance of these improvements, a paired-sample *t*-test was conducted across the 317 impact points. Comparing the proposed optimized framework (8.53 mm) against the unoptimized baseline (21.29 mm) and the intermediate state (without noise reduction, 12.36 mm) yielded a significant result (*p* < 0.001). This paired statistical analysis proves that the performance enhancement brought by the combined denoising and outlier weighting is robust across the entire structural field.

Furthermore, the paired-sample *t*-test comparing the proposed method against similarity metrics (Pearson Correlation, Cosine Similarity, DTW) also confirmed statistical significance (*p* < 0.001). Table 1 explicitly justifies the selection of Euclidean distance over alternatives such as DTW or correlation-based metrics through an optimal accuracy-efficiency trade-off. While DTW exhibits marginally superior absolute precision (7.99 mm), its high computational complexity results in a processing time of approximately 4.8 h (17,200.79 s) for the datasets. The proposed Euclidean distance metric processes the same datasets in just 4.5 min (270.24 s). Computationalefficiency has increased by nearly 63 times. Additionally, because the signals undergo Z-score normalization prior to matching, the Euclidean distance provides equivalent similarity assessments to Pearson correlation (8.53 mm vs. 8.34 mm) while requiring lower mathematical overhead for hardware implementation. In the context of aerospace SHM, sub-millimeter precision differences (a marginal 0.54 mm gap between DTW and Euclidean) fall entirely within acceptable physical engineering tolerances and environmental noise margins. By accepting a 0.54 mm accuracy trade-off compared to DTW, the Euclidean distance algorithm achieves the near-real-time responsiveness critical for embedded aerospace deployment.

Table 2 presents a literature comparison to explicitly demonstrate the research gaps addressed in this study. As shown in the table, the localization results are dependent on the test panel’s structural, the validation coverage, and the signal processing methods. Compared to previous methods used for detecting impact localization on composite panels, the use of an outlier weighting-based template matching method combined with sym5 wavelet threshold denoising demonstrates some advantage in enhancing both the computational efficiency and the overall reliability of impact localization.

Firstly, regarding signal complexity, it is evident that previous studies predominantly focused on thin, solid composite laminates with thicknesses ranging from 2 mm to 5 mm. In such thin plates, stress wave propagation is relatively straightforward. In contrast, this study tackles a 20 mm thick honeycomb sandwich panel. The multi-layer cellular core induces severe signal attenuation, mode conversion, and complex internal reverberations. This exceptionally high signal complexity is exactly why traditional methods struggle, and why the proposed combination of Sym5 wavelet threshold denoising and error outlier weighting is necessary to extract reliable localization features.

Secondly, regarding full-field validation, the existing literature frequently limits the impact test region to the central area of the panel, avoiding the boundaries where edge reflections severely distort the signals. Our method was tested over a 420 mm × 420 mm region on a 500 mm × 500 mm panel, achieving a 71% validation coverage while maintaining an average error of 8.53 mm. Compared to previous methods, this demonstrates an advantage in handling complex acoustic environments and enhancing the overall reliability of impact localization across the entire structure.

Regarding the practical implications for aerospace SHM deployment, the proposed framework demonstrates feasibility in both computational cost and hardware. The algorithm was executed on a system equipped with a 13th Gen Intel(R) Core (TM) i9-13900F processor and 64 GB of RAM. Because the template library is built offline prior to deployment, the online monitoring phase relies only on basic Sym5 wavelet filtering, Euclidean distance calculation, and outlier weighting. The processing speed of 0.85 s per impact measurement point is highly suitable for continuous flight monitoring, allowing onboard computers to instantly assess impacts (e.g., bird strikes or space debris) without inducing heavy computational latency. Furthermore, from a hardware deployment standpoint, FBG sensors are intrinsically advantageous for aerospace applications. Unlike traditional PZT sensors that require individual heavy wiring, multiple FBG sensors can be multiplexed along a single lightweight optical fiber. This reduces the parasitic weight and wiring complexity added to the spacecraft or aircraft. Coupled with their immunity to electromagnetic interference (EMI) in complex avionic environments, the proposed FBG-based weighted template matching system provides a practical solution for aerospace structures.

It is noteworthy that while the proposed method significantly reduces the global average error (from 21.29 mm to 8.53 mm) and effectively corrects large deviations, the accuracy for specific points experienced a slight decline. This phenomenon can be attributed to the complex wave propagation in honeycomb sandwich structures. For certain impact locations, the high-frequency components or specific sensor signals—which might be identified as ‘noise’ or ‘outliers’ by the algorithm—actually contain critical localization information. The outlier weighting strategy, while robust against large errors, essentially performs a weighted smoothing operation that may suppress these valid local distinct features. Additionally, wavelet denoising might filter out subtle but informative echoes along with environmental noise. However, the primary goal of this study is to enhance the robustness and reliability of the monitoring system. The significant reduction in maximum error and standard deviation confirms that the proposed method balances an acceptable reduction in local precision at specific nodes against a substantial gain in overall system stability.

The 16-sensor (4 × 4) uniform layout utilized in this study was established following a series of preliminary experimental trials. Initially, configurations with fewer sensors (e.g., 4 and 8) and irregular orientations (e.g., 14 sensors) were tested. However, these arrangements yielded poor and unstable localization accuracy. On a 20 mm thick honeycomb panel, which induces rapid stress wave attenuation, random or sparse orientations leave blind spots. Consequently, the uniform 4 × 4 grid was adopted to ensure overlapping sensitive zones and comprehensive full-field coverage. Furthermore, if fewer sensors were deployed, the mathematical foundation of the proposed localization algorithm would be compromised. The outlier-weighted template matching method relies heavily on statistical redundancy. To reliably calculate Z-scores and penalize anomalous edge signals, the algorithm requires a statistical majority to establish a baseline error distribution. A sparse sensor network (e.g., 4 to 8 sensors) fails to provide sufficient data points for accurate outlier detection, rendering the system vulnerable to local noise spikes. Thus, the 16-sensor network provides the necessary data redundancy to guarantee both physical sensing coverage and algorithmic robustness. Future research could explore formal optimization techniques (such as genetic algorithms) to find the absolute minimum sensor density required to maintain this statistical redundancy while reducing system weight for aerospace applications.

Overall, the denoising algorithm reduced both the average localization error and the maximum localization error, enhancing the accuracy of impact localization. Nevertheless, for certain results, the algorithm after denoising significantly enhanced localization accuracy, whereas for some other results, it conversely increased the localization error compared to the non-denoised version. This phenomenon is due to the honeycomb sandwich panel used in this study, which has a relatively intricate internal structure and produces multiple echoes. The denoising algorithm might have removed specific characteristics of some echoes, resulting in greater localization errors. Further research is needed to explore the effectiveness and stability of various denoising algorithms specifically for honeycomb sandwich panels.

## 6. Conclusions

By evenly distributing 16 FBG sensors on a 500 mm × 500 mm × 20 mm honeycomb sandwich panel within the constructed low-velocity impact monitoring system, low-velocity impact experiments were conducted, enhancing the low-velocity impact data for the honeycomb sandwich structure. An outlier-based template matching method was proposed that improves the localization accuracy through Sym5 wavelet denoising and weighting of error outliers, leading to more reliable identification. The proposed method demonstrates strong potential for reliable real-time sensing of low-velocity impacts on honeycomb sandwich panels and quick identification of impact locations. Achieving an average localization accuracy of 8.53 mm on a thick multi-layer structure, this study provides a valuable framework for improving the reliability and safety of honeycomb sandwich structures. It offers a methodology that could inform future impact localization in aerospace and aviation applications.

## Figures and Tables

**Table 1:** Statistical performance comparison of different similarity metrics on datasets with and without noise reduction (N = 317 impact points).

Similarity Metric	Avg. Error (mm)	Std. Dev (mm)	Variance (mm^2^)	95% CI (mm)	Processing Time (s)
Pearson Correlation	8.34	7.95	63.17	[7.46, 9.22]	349.09
Cosine Similarity	8.34	7.95	63.17	[7.46, 9.22]	280.28
Manhattan Distance	11.57	10.13	102.58	[10.50, 12.69]	275.11
Dynamic Time Warping (DTW)	7.99	8.09	65.38	[7.10, 8.89]	17,200.79
Euclidean Distance without outlier weighting	21.29	17.13	293.32	[19.47, 23.21]	258.34
Euclidean Distance without noise reduction	12.36	11.65	135.72	[11.08, 13.64]	261.71
**Euclidean Distance with noise reduction and outlier weighting**	**8.53**	**8.25**	**68.03**	**[7.62, 9.44]**	**270.24**

**Table 2:** Impact localization on composite panel results comparison.

Ref. N.	Structure Type & Thickness	Validation Coverage (Test Area/Total Area) (mm × mm)	N. of Sensors	Noise Mitigation/Algorithm	Avg. Error (mm)	Ref.
1	CFRP laminate, N/A	Not specified	8	Error outlier method	20.07	Ding et al., (2023) [60]
2	Quasi-isotropic composite structure, 4.7 mm	52%, (500 × 500/ 690 × 690)	4	Error outlier method	10.7	Shrestha et al., (2016) [50]
3	CFRP laminates, 3 mm	64%, (400 × 400/ 500 × 500)	9	BP neural network (PCA/FFT)	21.0	Wen et al., (2022) [49]
4	Aluminum alloy single-layer plate, 2.0 mm	44%, (400 × 400/ 500 × 500)	4	Morlet wave and Time reversal focusing model	20	Sai et al., (2014) [65]
5	Composite flat plate, 5.0 mm	Not specified	4	TDOA and MLP	9.17	Park et al., (2010) [66]
6	Woven composite plates, N/A	Not specified	5	TDOA	68	Hiche et al., (2010) [67]
7	CFRP laminates, 4.2 mm	30%, (150 × 180/ 300 × 300)	4	TDOA	10.0	Frieden et al., (2012) [68]
8	Composite flat plate, N/A	13%, (250 × 250/ 690 × 690)	4	BP neural network	8.2	Jang et al., (2012) [69]
9	Composite flat plate, 3.0 mm	49%, (400 × 400/ 650 × 650)	4	TDOA	9.74	Du et al., (2022) [70]
**This study**	**Honeycomb Sandwich, 20 mm**	**71%, (420 × 420/** **500 × 500)**	**16**	**Sym5 and Outlier Weighting**	**8.53**	

## Data Availability

The data sets presented in this article are not readily available, but results can be replicated under comparable experimental conditions. Requests to discuss replication of the results presented herein may be directed to the corresponding author.

## Abbreviations

The following abbreviations are used in this manuscript:

BVID	Barely Visible Impact Damage
TDOA	Time Difference of Arrival
FBG	Fiber Bragg Grating Sensors
SHM	Structural Health Monitoring
LEO	Low Earth Orbit
CFRP	Carbon Fiber Reinforced Plastic
FFT	Fast Fourier Transform
PCA	Principal Component Analysis
BP	Back Propagation
